# Protein, Creatine, and Dieting Supplements Among Adolescents: Use and Associations With Eating Disorder Risk Factors, Exercise-, and Sports Participation, and Immigrant Status

**DOI:** 10.3389/fspor.2021.727372

**Published:** 2021-10-13

**Authors:** Kethe Marie Engen Svantorp-Tveiten, Oddgeir Friborg, Monica Klungland Torstveit, Therese Fostervold Mathisen, Christine Sundgot-Borgen, Jan Harald Rosenvinge, Solfrid Bratland-Sanda, Gunn Pettersen, Jorunn Sundgot-Borgen

**Affiliations:** ^1^Department of Sports Medicine, The Norwegian School of Sport Sciences, Oslo, Norway; ^2^Department of Psychology, UiT -The Arctic University of Norway, Tromsø, Norway; ^3^Department of Sport Science and Physical Education, University of Agder, Kristiansand, Norway; ^4^Health and laboratory science, Department of Nursing, Østfold University College, Halden, Norway; ^5^Department of Sports, Physical Education and Outdoor Studies, University of South-Eastern Norway, Kongsberg, Norway; ^6^Department of Health and Care Sciences, UiT - The Arctic University of Norway, Tromsø, Norway

**Keywords:** dietary supplement, body image (MeSH), exercise (MeSH), mental health (MeSH), sport (MeSH), eating disorder, immigrant status, adolescent (MeSH)

## Abstract

**Objective:** This study aimed to estimate the number of weekly users of protein, creatine, and dieting supplements and to explore whether weekly use was related to eating disorder (ED) risk factors, exercise, sports participation, and immigrant status.

**Methods:** In total, 629 and 1,060 high school boys and girls, respectively, self-reported weekly frequency of protein, creatine, and dieting supplement use, and weight and shape concerns, appearance internalization and pressure, self-esteem, mental distress, physical activity level, exercise context, and the type and weekly frequency of sport played. Multiple hierarchical regression analyses were performed to investigate explanatory factors for supplement use.

**Results:** More boys than girls used protein and creatine supplements. Immigrant boys had more frequent use of all supplements than non-immigrant boys, and immigrant girls used creatine supplements more frequently than non-immigrant girls. In total, 23–40 and 5–6% of the variation in the weekly frequency of supplement use in boys and girls, respectively, was explained by immigrant status, ED risk factors, and exercise and sports participation. More frequent use of protein, creatine and dieting supplements in boys was significantly explained by more weight and shape concerns, fitness center exercise, and weight-sensitive sports participation. Depending on the type of supplement, more frequent use of supplements in girls was significantly explained by lower self-esteem, more engagement in weight-sensitive sports, and less engagement in general sport and exercise activities.

**Conclusion:** Weekly supplement use was common and more frequent among boys than girls. The weekly use of protein, creatine, and dieting supplements was related to ED risk factors, exercise and sports participation, and immigrant status in boys but not in girls.

## Introduction

The sports supplement industry has grown rapidly during the past decade (Marqual IT Solutions Pvt., Ltd. Research and Markets, 2019). Sports supplements represent a large group of dietary supplements used by most adult and adolescent high-performance athletes, and they may have an indirect or direct and assumed or documented effect on sports performance (Knapik et al., 2016). Among the most commonly used sports supplements in the general adolescent population are protein, creatine, and dieting supplements, such as fat burners and appetite suppressants (Eisenberg et al., 2012; Tiwari, 2020), and are used to achieve a more muscular and lean appearance (Knapik et al., 2016).

The long-term health effects of protein and creatine supplements are not well-known (Pope et al., 2014). Even if properly administered use of non-contaminated creatine or protein supplements in experimental studies has not been linked to health risks in adults (Jagim et al., 2018), research suggests that use in a natural setting is related to several negative health outcomes in adolescents (Or et al., 2019; Tiwari, 2020); this is partially related to a high proportion of contaminated products (Walpurgis et al., 2020), leading to severe and unintended health consequences (Hoffman et al., 2008; Pope et al., 2014). In addition, the use of protein and creatine supplements may also act as a gateway to future steroid use (Backhouse et al., 2013; Hurst et al., 2020). Consequently, adolescents should not use supplements to enhance performance or appearance (Bergeron et al., 2015).

Previous studies involving older adolescents in the general population found that up to 50% of boys (Hoffman et al., 2008; Eisenberg et al., 2012; Yager and McLean, 2020) and 20% of girls (Eisenberg et al., 2012) had ever used or used protein supplements during the past year. Meanwhile, the estimated overall proportion of general adolescents currently or previously using creatine supplements varies from 0.6 to 22.2% in boys and 0.6 to 3% in girls (Hoffman et al., 2008; Jagim et al., 2018; Miech et al., 2020; Yager and McLean, 2020). A recent study also found the 30-day prevalence of diet pill use in late adolescent boys and girls to be 1.5 and 1.7%, respectively (Miech et al., 2020).

Several factors predict the use of protein and creatine supplements; among these is gender (Eisenberg et al., 2012). Ethnicity is suggested as another factor possibly explaining supplement use as a means of changing appearance (Eisenberg et al., 2012; Yager and McLean, 2020). Males of non-white ethnicities may experience more drive for muscularity (Swami, 2016) and body dissatisfaction and engage in more extreme body change strategies (Ricciardelli et al., 2007), while girls of some non-white ethnicities may experience less body dissatisfaction than white ethnicities (Kimber et al., 2015). In addition, boys of Asian ethnicity are found to use more “muscle enhancing” supplements than non-Asians (Eisenberg et al., 2012). However, existing findings are conflicting and inconclusive (Eisenberg et al., 2012; Kimber et al., 2015; Yager and McLean, 2020). Therefore, investigating immigrant status relative to supplement use, as opposed to ethnicity, may contribute further to the understanding of supplement use in adolescents. The experiences of first or second-generation immigrants could also potentially differ from those of later-generation immigrants (Forbes, 2010; Swami, 2016). This may partially be explained by how sociocultural characteristics fade over time with integration and how integrated individuals perceive themselves as more powerful and with less stress related to identity and masculinity (Liu and Concepcion, 2010). Other factors which may explain protein, creatine, and dieting supplement use are exercise and sports participation. Previous studies have examined the association between protein supplement use and sports participation, finding that a greater number of sports played (Yager and McLean, 2020), but not participating in a sports club in general (Eisenberg et al., 2012), was associated with protein supplement use. Furthermore, the association between supplement use and participation in weight training is conflicting (Yager and O'Dea, 2014; Yager and McLean, 2020). The role of exercise context and frequency of playing different types of sports in relation to protein, creatine, and dieting supplement use has not been investigated to date in the general adolescent population. Different sports and exercise contexts may be perceived as objectifying appearance (Sundgot-Borgen et al., 2021) and place a greater emphasis on muscularity and strength, which may lead to the use of supplements advertised as being muscularity and performance-enhancing (Sandvik et al., 2018).

Most studies investigating supplement use in adolescents have observed athletes, and less knowledge exists regarding such use in the general adolescent population, especially in girls. Previous studies have found that body image, self-esteem, and internalization of appearance ideals are associated with muscle-building behaviors in general, where supplement use is incorporated (Smolak et al., 2005; Smolak and Stein, 2010; Rodgers et al., 2020). However, only two studies have investigated the association between body image, exercise or sports participation, and protein supplement use in regular adolescents (Yager and O'Dea, 2014; Yager and McLean, 2020), finding that use is explained by body dissatisfaction (Yager and O'Dea, 2014), muscularity beliefs, number of sports activities, and weight training (Yager and McLean, 2020). None of these studies included girls; however, one older study including both girls and boys found that supplement use in general was associated with body image and appearance idealization but not exercise and sports participation (Field et al., 2005). Furthermore, the inclusion of measures of a wider range of exercise and sports characteristics, such as physical activity level, exercise contexts, multiple types of sport activities, and the frequency of sports participation (Yager and McLean, 2020), have been requested. Creatine is considered one of the most potent legal sports supplements on the market, with a documented effect on high-intensity and short-duration physical performance (Hall and Trojian, 2013). Therefore, its use could be solely explained by exercise and sports participation and the desire for enhanced performance. No studies have investigated explanatory factors for creatine supplementation in general adolescents. However, recent studies including adults found that ergogenic supplement use (such as creatine) was explained by eating disorder (ED) cognitions and behaviors in both males and females (Nagata et al., 2020a); moreover, users had more positive attitudes toward doping (Hurst et al., 2019). As such, knowledge of whether and how creatine supplement use is associated with ED risk factors and exercise behaviors in adolescents is warranted.

The association between dieting supplement use and psychological factors is known in girls (Wang et al., 2014), yet knowledge on this matter in adolescent boys is sparse. Although several high-quality studies have reported on the annual or lifetime prevalence of protein, creatine, and dieting supplement use, no studies in the past decade have reported the proportion of adolescents using these supplements weekly or more often, this information is important for several reasons. More frequent users may differ from occasional users since they may be more driven toward enhancing performance and appearance. The sports supplement industry is growing, yet knowledge about the “severity” of use in adolescents is limited. Hence, knowledge regarding whether more frequent use is associated with more unfavorable outcomes is unknown. Such knowledge may indicate whether preventive efforts should be aimed at frequent users or users in general.

Therefore, this study aimed (1) to estimate the proportion of late adolescent boys and girls using protein, creatine, and dieting supplements weekly and (2) to investigate whether ED risk factors, exercise and sports participation, and immigrant status explain the variance in the weekly frequency of protein, creatine, and dieting supplement use.

## Materials and Methods

### Recruitment

**Figure 1:**
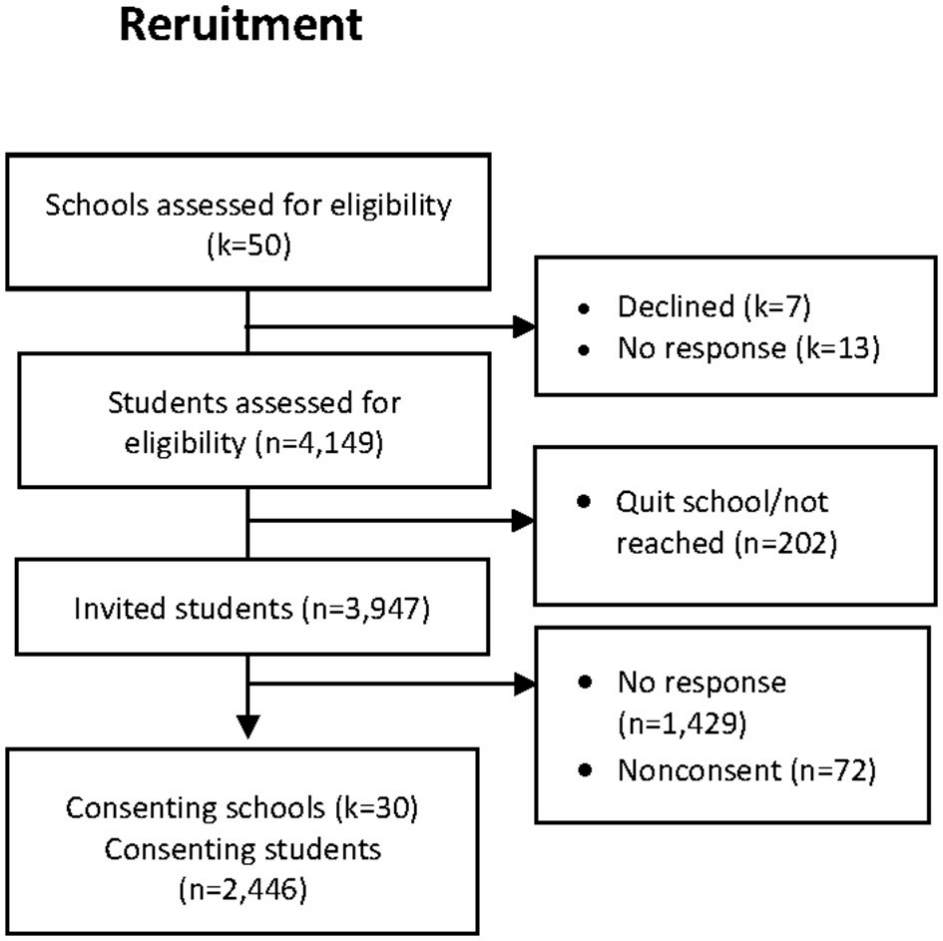
Participant and school recruitment. *k*, number of schools; *n*, number of students.

The current cross-sectional study included baseline assessments from an intervention study (Sundgot-Borgen et al., 2018) aiming to promote a positive body image and to reduce the risk of ED development (The healthy body image program; HBI) in 16–18-year-old high school adolescents. In the fall of 2016, all public and private high schools in the Oslo and Akershus counties were asked to participate in the study. The request, together with the study information, was presented to the school principals and administrators. Of the 50 eligible schools, 30 agreed to participate. A meeting was arranged at each school, and all students were thoroughly informed of the study aims and the implications of participation. The students subsequently received information and informed consent letters together with the questionnaires. Figure 1 shows the school and participant flow. Of the 3,947 invited students, 2,446 gave their consent to participate in the study. In total, 757 students did not answer questions about supplement use, resulting in 1,689 students being included in the cross-sectional analyses.

### Measures

#### Demographics and Body Mass Index

Participant age (years), body weight (kg), body height (cm), and immigrant status were assessed by self-report. Immigrant status was assessed according to the definition given by Statistics Norway regarding a first- or second-generation immigrant (two foreign-born parents) and did not include individuals who were foreign-born, had two foreign-born parents, or adopted by Norwegian-born parents. Self-reported body weight and height were used to calculate body mass index (BMI).

#### Protein, Creatine, and Dieting Supplement Use

Usage of protein, creatine, and dieting supplements were the main outcome variables. Outcomes were measured with self-developed questions on the weekly frequency of supplement use. Protein and creatine supplements included powders usually mixed with fluid. Dieting supplements included products consumed to aid weight or fat loss (i.e., fat burners, pills, and powders). Questions were scored on a five-point Likert scale from 1 (never) to 5 (daily). All students who reported using supplements at least one time per week were defined as “weekly users.”

#### Eating Disorder Risk Factors

*Weight and shape concerns* were measured with an empirically derived brief version of the Eating Disorder Examination Questionnaire (Friborg et al., 2013). The scale consists of 11 items scored on a Likert scale ranging from 0 to 6, where a higher score reflects greater weight and shape concerns. Cronbach's alpha in this sample was 0.91 and 0.94 for boys and girls, respectively.

*The sociocultural attitudes toward appearance questionnaire*-*4* (Schaefer et al., 2015) was used to assess societal and interpersonal aspects of appearance ideals. Three subscales—thin/low body fat internalization (Thin internalization), muscular/athletic internalization (Muscular internalization), and perceived pressure from media (Media pressure)—were included in this study. Items were scored on a five-point Likert scale ranging from “strongly disagree” to “strongly agree,” where a higher score indicates a higher degree of internalization or perceived pressure. Cronbach's alpha for the three subscales ranged from 0.85 to 0.94 in boys and 0.91 to 0.95 in girls.

*The Rosenberg self-esteem scale* (Rosenberg, 1965) measures global self-worth and was used as a measure of self-esteem. The scale consists of 10 items scored on a five-point Likert scale ranging from 1 to 4, where a higher score represents higher self-esteem. Cronbach's alpha was 0.90 and 0.92 in boys and girls, respectively.

*Mental distress* was measured by the Hopkins Symptom Checklist 10, which assesses symptoms of anxiety and depression (Strand et al., 2003). The scale comprises 10 items scored on a five-point Likert scale ranging from 0 to 4, where higher scores represent more mental distress. Cronbach's alpha for this present sample was 0.89 and 0.90 in boys and girls, respectively.

#### Exercise- and Sports Participation

*Physical activity level* was assessed by asking the participants how many hours and minutes they spent being physically active during a normal week. Physical activity was defined within the questionnaire as “any activity making you warm and slightly breathless (e.g., physical education, activities with your family, active transportation (i.e., walking and biking to school, exercise, sports, and self-organized activities).”

*Exercise context* was assessed by participants reporting whether they were active members and exercising at a fitness center or in organized sports using response categories. The questions were recoded into two dichotomous variables: fitness center exercise (0 = no, 1 = yes) and organized sport participation (0 = no, 1 = yes).

*The type and weekly frequency of sports participation* were assessed by a self-developed questionnaire listing several different types of exercise/sports and asking how many times per week they engaged in different exercise or sport activities. Participants scored each activity on a four-point Likert scale ranging from never to several times per week. To reduce the number of correlated sports and exercise variables and create meaningful groups of exercise and sport activities, a principal component analysis with varimax rotation was performed. The analysis yielded three components with Eigenvalues > 1.0. This three-factor solution aligned with previously suggested groupings of weight-sensitive sports (Sundgot-Borgen et al., 2013): (1) weight class and gravitational sports (martial arts, CrossFit, fitness, powerlifting, weightlifting and power sports), (2) aesthetic sports (gymnastics, diving, figure skating, dance, and yoga), and (3) general exercise and sports activities (endurance exercise, resistance exercise, and ball game sports). Factor structure, loadings, and explained variance are provided in the Supplementary Table 1.

### Statistical Analyses

All analyses were performed using IBM SPSS statistics version 24. Continuous variables are presented as means and standard deviation, while categorical data are presented as the number of observations (*n*) and proportions (%). Between-group differences in demographic psychometric and exercise variables were tested using an independent sample *t*-test and Fisher's exact test. The Mann-Whitney *U*-test was performed to investigate overall differences between boys and girls regarding protein, creatine, and dieting supplement use. Cohen's d (d) and odds ratios were used as effect sizes for parametric and non-parametric comparisons, respectively. The alpha level was set to *p* = 0.05. Hierarchical multiple linear regression analysis was performed to investigate potential explanatory variables for protein supplement use, creatine supplement use, and dieting supplement use. Bootstrapping was performed to handle non-normal residual score distributions and was performed using a wild sampling method with unstandardized residuals as value variables. The number of bootstrap samples was set to 2000 with bias-corrected and accelerated 95% confidence intervals. The variance inflation factor (≤5.0), condition number (<10), and variance decomposition proportion (>80%) were investigated, and no violations of cut points existed (Kim, 2019). All variables were standardized to Z scores to ease interpretation and to obtain standardized beta weights from the bootstrapped analysis.

The dependent variables were weekly protein, creatine, and dieting supplement use. The independent variables were included in three steps as follows. Step (1) immigrant status with BMI as the adjustment variable. Step (2) weight and shape concerns, thin internalization, muscular internalization, media pressure, self-esteem, and mental distress. Finally, Step (3) physical activity, sports, and exercise variables: physical activity hours/week, organized sports participation (no/yes), fitness center exercise (no/yes), weight class and gravitational sports, aesthetic sports, and general sports and exercise. The results are presented as the standardized beta weight and 95% confidence intervals. All analyses were stratified by gender.

### Ethics

The study met the intent and requirements of the Health Research Act and the Helsinki Declaration regarding informed consent and unconditional withdrawal and was approved by the Regional Committee for Medical and Health Research Ethics (2016/142). This work was supported by the DAM foundation (2016/FO76521), through the Norwegian Woman's Public Health Association (H1/2016). A commercial sponsor (TINE AS) supported the study after the study protocol was published (Sundgot-Borgen et al., 2018) but was not involved in data collection, data analysis, or the writing of the present article.

## Results

Students who did not answer questions about supplement use were classified as dropouts. They were not included in the analysis. Dropouts did not differ from respondents in demographic or psychometric measures.

Demographic characteristics, psychometric scores, and exercise and sport participation in girls and boys are presented in Table 1. Overall, boys used more protein (*U* = 265514.50, *Z* = −10.73, *p* = <0.001) and creatine (*U* = 849429.50, *Z* = −10.09, *p* = <0.001) supplements compared to girls. No between-group differences were observed for dieting supplement use (Table 2). Girls who used supplements consumed these less often than boys who used supplements, and boys were more likely than girls to use two or more supplements in combination (Table 2). Immigrant boys consumed protein (*U* = 267184.50, *Z* = 3.58, *p* = <0.001), creatine (*U* = 25588.00, *Z* = 3.17, *p* = 0.002) and dieting (*U* = 23901.50, *Z* = 2.05, *p* = 0.040) supplements more frequently than non-immigrant boys. Immigrant girls consumed creatine supplements more frequently than non-immigrant girls (*U* = 66960, *Z* = 2.56, *p* = 0.010).

**Table 1:** Descriptive characteristics for boys and girls presented as the mean (standard deviation) or number of observations (percentage) with Cohen's d (d) or odds ratio (OR) as effect size (ES) for scale and dichotomous variables, respectively.

	**Boys** **(*n* = 629)**	**Girls** **(*n* = 1,060)**	***p***	**ES**
Age	16.76 (0.48)	16.78 (0.48)	0.437	
BMI (kg/m^2^)	21.78 (2.80)	21.38 (2.84)	**0.005**	0.14^d^
Immigrants, *n* (%)	82 (13.0)	140 (13.2)	0.951	
Weight and shape concerns	1.0 (1.6)	2.5 (1.7)	**<0.001**	1.05^d^
Thin internalization	2.5 (0.95)	3.3 (1.1)	**<0.001**	0.36^d^
Muscular internalization	3.2 (1.1)	3.0 (1.1)	**<0.001**	0.20^d^
Media pressure	2.1 (1.2)	3.2 (1.2)	**<0.001**	0.88^d^
Self-esteem	32.8 (6.0)	29.1 (6.2)	**<0.001**	0.61^d^
Mental distress	1.6 (0.6)	2.2 (0.7)	**<0.001**	0.81^d^
Physical activity (hours/week)	8.44 (6.11)	6.84 (5.19)	**<0.001**	0.28^d^
Fitness center exercise, *n* (%)	346 (55.0)	441 (41.6)	**<0.001**	1.72^OR^
Organized sports participation, *n* (%)	333 (52.9)	406 (38.3)	**<0.001**	1.81^OR^
Weight class and gravitational sports, *n* (%)	243 (38.6)	271 (25.6)	**<0.001**	1.83^OR^
Aesthetic sports, *n* (%)	61 (9.7)	348 (32.8)	**<0.001**	0.22^OR^
General sports and exercise, *n* (%)	560 (89.0)	855 (80.7)	**<0.001**	1.95^OR^

*BMI, body mass index;*

d *Cohen's d;*

OR *odds ratio. Significant differences (p values) are highlighted in bold*.

**Table 2:** Supplement use, frequency of use and number of supplements used in boys and girls. presented as the number of observations (%).

	**Boys** **(*n* = 629)**	**Girls** **(*n* = 1,060)**	***p***	**OR**
**Weekly protein supplement use**, ***n*** **(%)**	**185 (29.4)**	**101 (9.5)**	**<0.001**	**3.96**
**Frequency of use ^a^**				
1–2 days/week	70 (37.8)	54 (53.5)	**0.013**	**0.53**
3–4 days/week	65 (35.1)	27 (26.7)	0.185	
5–6 days/week	19 (10.3)	11 (10.9)	0.843	
Everyday	31 (16.8)	9 (8.9)	0.059	
**Weekly creatine supplement use**, ***n*** **(%)**	**105 (16.7)**	**32 (3.0)**	**<0.001**	**6.44**
**Frequency of use ^a^**				
1-2 days/week	18 (17.1)	15 (46.9)	**0.002**	**0.23**
3-4 days/week	44 (41.9)	12 (37.5)	0.687	
5-6 days/week	11 (10.5)	3 (9.4)	1.000	
Everyday	32 (30.5)	2 (6.2)	**0.005**	**6.58**
**Weekly dieting supplement use**, ***n*** **(%)**	**50 (7.9)**	**74 (7.0)**	0.500	
**Frequency of use ^a^**				
1–2 days/week	16 (32.0)	57 (77.0)	**<0.001**	**0.14**
3–4 days/week	14 (28.0)	11 (14.9)	0.109	
5–6 days/week	5 (10.0)	1 (1.4)	**0.039**	**8.11**
Everyday	15 (30.0)	5 (6.8)	**0.001**	**5.91**
**Number of supplements/aids used**				
Using one supplement/aid	102 (50.7)	128 (78.5)	**<0.001**	**0.28**
Using two supplements/aids	59 (29.4)	26 (16.0)	**0.003**	**2.19**
Using all three supplements/aids	40 (19.9)	9 (5.5)	**<0.001**	**4.25**

a *Frequency of use among users.* *Odds ratio (OR) is shown for significant between group differences was tested by applying Fishers exact test. Significant differences (p values) are highlighted in bold*.

The hierarchical multivariable regression analyses for weekly frequency of protein, creatine, and dieting supplement use in boys and girls are shown in Tables 3, 4, respectively. In boys, the first step including immigrant status, adjusted for BMI, explained 1–2% of the variance in protein and creatine supplement use and 9% of the variance in dieting supplement use. The inclusion of psychometric measures in step 2 of the analyses significantly improved the explained variance to 13, 11, and 20% for weekly frequency of protein, creatine, and dieting supplement use, respectively. The inclusion of exercise and sport participation variables in the final step doubled the explained variance for both protein, creatine, and dieting supplement use.

**Table 3:** Bootstrapped hierarchical linear regression analysis of variables explaining the variance in boys' (*n* = 629) weekly (0–7) use of protein, creatine, and dieting supplements, respectively.

	**Protein**			**Creatine**			**Dieting**		
	**Step 1**	**Step 2**	**Step 3**	**Step 1**	**Step 2**	**Step 3**	**Step 1**	**Step 2**	**Step 3**
	**ß _95%CI_**	**ß _95%CI_**	**ß _95%CI_**	**ß _95%CI_**	**ß _95%CI_**	**ß _95%CI_**	**ß _95%CI_**	**ß _95%CI_**	**ß _95%CI_**
Immigrant status (yes/no)	**0.13**_049|0.212_	**0.12**_034|0.200_	0.08 _−0.009|0.159_	**0.10 _021|0.190_**	0.08 _−0.002|0.169_	0.03 _−0.050|0.121_	**0.10._022|0.184_**	0.05 _−0.034|0.126_	−0.01_−0.076|0.090_
Weight and shape concerns		**0.20 _100|0.312_**	**0.18 _069|0.289_**		**0.26 _138|0.363_**	**0.22 _099|0.331_**		**0.42 _256|0.580_**	**0.38 _225|0.543_**
Thin internalization		−0.08 _−0.177|0.014_	−0.03 _−0.128|0.069_		**−0.14 _−0.234|−0.035_**	**−0.11 _−0.206|−0.011_**		0.04 _−0.032|0.103_	0.04 _−0.034|0.102_
Muscular internalization		**0.31 _227|0.385_**	**0.19 _0.092|0.297_**		**0.21 _118|0.297_**	**0.12 _018|0.220_**		−0.06 _−0.111|−0.002_	−0.08 _−0.155|0.006_
Media pressure		0.05 _−0.076|0.095_	−0.01 _−0.083|0.088_		0.04 _−0.042|0.123_	0.03 _−0.057|0.112_		0.02 _−0.055|0.113_	−0.01 _−0.083|0.082_
Self-esteem		0.01 _−0.080|0.107_	0.05 _−0.042|0.148_		0.04 _−0.062|0.143_	0.08 _−0.022|0.180_		−0.07 _−0.177|0.036_	−0.03 _−0.134|0.075_
Mental distress		0.01 _−0.087|0.95_	0.02 _−0.076|0.110_		0.01 _−0.084|0.117_	0.02 _−0.079|0.131_		−0.05 _−0.161|0.078_	−0.03 _−0.140|0.093_
Physical activity (hours/week)			−0.04 _−0.124|0.052_			0.02 _−0.078|0.099_			−0.04 _−0.132|0.056_
Fitness center exercise (yes/no)			**0.20 _0.122|0.276_**			**0.09 _011|0.168_**			**0.10 _024|0.170_**
Organized sports participation (yes/no)			−0.01 _−0.101|0.083_			−0.04 _−0.132|0.048_			0.08 _−0.011|0.172_
Weight class/gravitational sports			**0.25_0.147 |0.360_**			**0.31 _196|0.425_**			**0.19 _088|0.290_**
Aesthetic sports			0.07 _−0.016|0.151_			**0.15 _056|0.249_**			**0.36 _244|0.474_**
General sports and exercise			0.01 _−0.089|0.104_			−0.01 _−0.095|0.092_			−0.04 _−0.140|0.064_
BMI (kg/m^2^)	0.06 _−0.001|0.134_	−0.03 _−0.104|0.045_	−0.08 _−0.147|0.002_	−0.04._035|0.096_	−0.05 _−0.129|0.024_	**−0.09 _−0.170|−0.005_**	0.03 _−0.043|0.095_	**−0.10 _−0.183|−0.023_**	**−0.12 _−0.204|−0.041_**
*F*	7.50^a^	13.08^a^	15.837^a^	3.93^a^	8.85^a^	14.71^a^	3.77	16.49^a^	18.60^a^
Adjusted *R*^2^	0.02^b^	0.13^b^	0.25^c^	0.01^c^	0.11^b^	0.23^b^	0.09^c^	0.20^b^	0.40^b^

*BMI, body mass index.*

a *p = < 0.001,*

b *p R^2^ change = <0.001,*

c *p R^2^ change = <0.05; ß, standardized coefficient; 95%CI, 95% confidence interval.*

*Significant (p ≤ 0.05) beta weights and CI are highlighted in bold. All analyses were adjusted for BMI*.

**Table 4:** Bootstrapped hierarchical linear regression analysis of variables explaining the variance in girls' (*n* = 1,060) weekly (0–7) use of protein, creatine, and dieting supplements, respectively.

	**Protein**			**Creatine**			**Dieting**		
	**Step 1**	**Step 2**	**Step 3**	**Step 1**	**Step 2**	**Step 3**	**Step 1**	**Step 2**	**Step 3**
	**ß _95%CI_**	**ß _95%CI_**	**ß _95%CI_**	**ß _95%CI_**	**ß _95%CI_**	**ß _95%CI_**	**ß _95%CI_**	**ß _95%CI_**	**ß _95%CI_**
Immigrant status (yes/no)	0.05 _−0.026|0.118_	0.05 _−0.016|0.124_	0.04 _−0.036|0.116_	**0.10 _002|0.191_**	0.09 _−0.002|0.172_	0.08 _−0.018|0.168_	−0.03 _−0.092|0.020_	−0.03 _−0.083|0.018_	−0.04._096|0.013_
Weight and shape concerns		−0.01 _−0.140|0.144_	−0.01 _−0.174|0.105_		0.05_−0.167|0.268_	0.05 _−0.174|0.258_		**0.21 _015|0.372_**	**0.20 _007|0.368_**
Thin internalization		−0.06 _−0.153|0.034_	−0.05._186|0.031_		−0.10_0.245|0.038_	−0.09 _−0.232|0.057_		−0.04 _−0.158|0.034_	−0.03 _−0.149|0.118_
Muscular internalization		0.13_0.060|0.200_	0.06 _−0.030|0.131_		0.02 _−0.045|0.075_	−0.04 _−0.116|0.031_		**0.07 _007|0.114_**	0.05 _−0.024|0.118_
Media pressure		−0.11 _−0.181|0.039_	−0.10 _−0.067|0.068_		−0.05 _−0.120|0.028_	−0.03 _−0.104|0.045_		−0.08 _−0.105|0.079_	−0.07 _−0.091|0.093_
Self-esteem		**−0.17 _−0.258|−0.083_**	**−0.17 _−0.258|−0.080_**		−0.16 _−0.265|−0.054_	**−0.16 _−0.266|−0.047_**		−0.08 _−0.172|0.015_	−0.08 _−0.174|0.021_
Mental distress		−0.05 _−0.133|0.022_	−0.06 _−0.152|0.013_		−0.06 _−0.162|0.041_	−0.07 _−0.169|0.031_		−0.01 _−0.105|0.080_	−0.03 _−0.116|0.068_
Physical activity (hours/week)			0.07 _−0.019|0.158_			0.02 _−0.075|0.122_			0.05 _−0.025|0.122_
Fitness center exercise (yes/no)			0.01 _−0.050|0.080_			0.02 _−0.069|0.093_			0.04 _−0.031|0.111_
Organized sports participation (yes/no)			−0.05 _−0.110|0.031_			0.02 _−0.075|0.093_			0.01 _−0.055|0.086_
Weight class/gravitational sports			**0.14 _060|0.237_**			**0.16 _040|0.284_**			0.08 _−0.023|0.201_
Aesthetic sports			0.04 _−0.021|0.108_			**0.09 _008|0.174_**			0.06 _−0.005|0.129_
General sports and exercise			0.01 _−0.072|0.086_			−0.02 _−0.102|0.067_			**−0.09 _−0.173|−0.016_**
BMI (kg/m^2^)	−0.04 _−0.098|0.016_	0.04 _−0.103|0.028_	−0.04 _−0.113|0.036_	−0.03 _−0.079|0.023_	−0.04 _−0.113|0.028_	0.04 _−0.109|0.035_	**0.09 _035|0.153_**	0.04 _−0.036|0.103_	0.04 _−0.035|0.107_
*F*	5.58^a^	4.32^a^	4.82^a^	5.58^a^	4.23^a^	4.82^a^	5.13^b^	7.37^a^	5.953^a^
Adjusted *R*^2^	0.03^c^	0.05^c^	0.05	0.01^d^	0.03^d^	0.06^d^	0.05^d^	0.06^d^	0.06^d^

*BMI, body mass index.*

a *p = < 0.001,*

b *p = < 0.01,*

c *p = < 0.05,*

d *p R^2^ change = < 0.001; ß, standardized coefficient; 95 % CI, 95% confidence interval.*

*Significant (p ≤ 0.05) beta weights and CI are highlighted in bold. All analyses were adjusted for BMI*.

In girls, the first step including immigrant status only explained 1, 3, and 5 % of the variance in the weekly frequency of protein, creatine, and dieting supplement use, respectively. Including psychometric variables in the second step and exercise and sport participation variables in the final step only marginally improved the explained variance.

## Discussion

As expected, boys were almost four times more likely to report weekly use of protein and creatine supplements and were more frequent users than girls. Interestingly, immigrant boys were more frequent users of all supplements, and immigrant girls used creatine supplements more frequently than their non-immigrant counterparts. The use of protein, creatine, and dieting supplements was more strongly associated with ED risk factors as well as exercise and sport participation in boys than in girls.

### Boys

Boys who were more frequent users of protein, creatine, and dieting supplements could be characterized by higher weight and shape concerns, as fitness center exercisers, and as more active in weight-class and gravitational sports. Unsurprisingly, more frequent use of protein and creatine supplements was explained by higher muscular internalization, and more frequent use of creatine supplements was associated with less thin appearance ideal internalization. Experiencing lower levels of thin appearance internalization might be a positive outcome regarding ED development. However, results from previous research conclude that increased internalization of muscularity without thinness internalization is predictive of more severe muscle dysmorphia in males (Klimek et al., 2018). More frequent use of dieting supplements was explained by higher weight and shape concerns and more frequent participation in weight-class and gravitational and aesthetic sports was expected since disordered eating (DE) is more prevalent among athletes in these sports (Sundgot-Borgen et al., 2013). Our results suggest that more frequent use of protein, creatine, and dieting supplements in boys is associated with factors known to increase the risk of developing ED considering the higher weight and shape concerns, muscular internalization (Taylor, 2016; Schaefer et al., 2017), and engagement in exercise and sport participation emphasizing appearance and leanness (Sundgot-Borgen et al., 2013, 2021). The current study reflects and expands previous research findings that the use of protein and creatine supplements is associated with higher muscle-oriented body dissatisfaction (Yager and O'Dea, 2014) and drive for muscularity in adolescent boys (Yager and McLean, 2020), as well as ED pathology in males (Nagata et al., 2020a). In contrast, a roughly comparable study concluded that protein supplement use was only associated with the number of sports played and weight training and not body image (Yager and McLean, 2020). The current study expands current knowledge by indicating that one should consider the “severity” of supplement use rather than “any use.” Furthermore, the findings indicate that it may be wise to give attention to boys who frequently consume supplements regularly since this may indicate a problematic relationship with body image and exercise.

### Girls

More frequent use of protein- and creatine supplements was associated with having slightly lower self-esteem, higher muscular internalization (not creatine users), and performing weight-sensitive sports. Dieting supplement use in girls was, as expected, associated with more weight and shape concerns and less participation in general sports and exercise activities. Although the association was weak, self-esteem is considered a fundamental psychological state important for mental health and quality of life later in life and in the transition to adulthood (Boden et al., 2008). Interestingly, performing general endurance and strength exercises and ball game activities stood out as potential protective factors for dieting supplement use in girls. This is not surprising considering that general physical activity and exercise affect physical fitness, body composition, and physical self-efficacy, which in turn predict increased body esteem and global self-esteem in girls (Gothe et al., 2021). Importantly, immigrant status, ED risk factors, and exercise and sport participation variables only accounted for 5–6% of the variation in protein, creatine, and dieting supplement use in girls, thus more than 94% of the variance is explained by other non-measured factors or measurement error. Comparable literature is lacking. However, the findings of the current study are in contrast to one recent study concluding that the use of ergogenic supplements is associated with ED pathology in adult women (Nagata et al., 2020a). The results of the current study add novel information about the association between supplement use and ED risk factors and exercise in adolescent girls. This study further refines current knowledge about the association between muscle-building strategies and DE in girls (Rodgers et al., 2020) by asserting that protein, creatine, and dieting supplement use in general may not be as relevant in the context of DE without considering motives for use (i.e., for appearance or performance reasons).

### Immigrant Status

The significant explained variance from immigrant status regarding protein and creatine supplement use vanished when sport and exercise variables were entered into the analyses. The results of this study suggest that the difference in use among immigrant and non-immigrant boys found initially may be partially explained by differences in sport and exercise participation. This finding expands current research concluding that muscle-building behaviors differ between ethnicities (Eisenberg et al., 2012; Nagata et al., 2020b); this finding indicates that differences in sport and exercise participation may explain the previously observed ethnic differences in supplement use. The significant explained variance from immigrant status regarding dieting supplements disappeared when ED risk factors were included in the analyses, indicating that the difference between immigrant and non-immigrants use of dieting supplements found in our study could be partially explained by differences in psychological measures (e.g., higher weight and shape concerns) among immigrant boys compared to non-immigrant boys. That creatine supplement use was more common among immigrant girls than non-immigrant girls is a novel finding not reported in previous studies. However, the association between immigrant status and creatine supplement use was weak and not well explained by the variables included in the current study.

### Number of Users

The findings of this study may indicate that protein and creatine supplement use is more common in Norwegian adolescents compared to American and Australian adolescents (Miech et al., 2020; Yager and McLean, 2020). This may raise the question of whether supplement use has increased over time (Field et al., 2005; Eisenberg et al., 2012) to a level comparable to what adult elite athletes used 15 years ago (Sundgot–Borgen et al., 2003). Previous research in adolescents has highlighted that adolescents do not hold a sufficient amount of knowledge regarding the proper use and potential health consequences of sports supplements (Whitehouse and Lawlis, 2017). Additionally, there was a notably high number of infrequent creatine supplement users in the current sample. For creatine supplementation to be effective, the supplement must be consumed daily in combination with exercise and not consumed as a “pre-workout supplement” (Hall and Trojian, 2013). This finding may suggest that knowledge about proper use may be poor in both genders. Increasing knowledge about proper use may be warranted. However, there may be several other explanations for infrequent use, such as lack of finances to purchase supplements or the lack of routines making it hard to remember to consume creatine daily. The large proportion of supplement users in the current sample raises further concerns due to potential negative health consequences (Or et al., 2019; Tiwari, 2020), unknown long-term health effects (Pope et al., 2014), high risk of consuming contaminated products (Hoffman et al., 2008; Pope et al., 2014; Walpurgis et al., 2020). and future steroid use (Backhouse et al., 2013; Hurst et al., 2020).

### Strengths and Limitations

The strengths of this study include large sample size and the inclusion of both girls and boys. The study also assessed both protein and creatine and dieting supplement use, as well as more frequent use than previous research. The inclusion of several psychological measures and assessment of physical activity level, exercise contexts, and different types and weekly frequency of sports participation, as suggested by previous studies, is also of note. However, the proportion of non-responders was large, and the sample of girls who used supplements was small, increasing the risk of type II error for those results. Another limitation is the use of a cross-sectional design limiting the ability to draw conclusions regarding causality between the variables examined. Furthermore, we did not include measures of other dietary supplements that may be less associated with body image and ED outcomes (e.g., vitamin supplements) (O'Dea, 2003). Therefore, the findings cannot be generalized to other dietary supplements. This study did not include measures regarding the duration of or reasons for supplement use; therefore, reasons for use can only be considered speculations. It is likely that long-time users differ from short-time users in psychological, sport, and exercise variables. In addition, the study did not assess competitive level of sport participation which limits the ability to investigate the influence of competitive sport participation on the results. Finally, only measuring physical activity level by subjective measures as well as using self-reported body height and weight provides a well-known bias.

### Implications and Future Research

Prevention of supplement use in adolescents is warranted due to the high proportion of young users in the current study. Prevention programs should aim to increase knowledge about supplements and resilience and literacy toward supplement advertising and marketing. Prevention efforts are also suggested to be especially aimed at adolescent boys exercising at fitness centers and those participating in weight-sensitive sports. Increasing knowledge about exercise adaptation, nutrition, and supplement use in adolescents among trainers in fitness centers and sports teams may also prevent adolescent use of supplements. Policy change and legislation are also needed to better regulate the industry and include statutory third-party testing of supplements to reduce the risk of large proportions of contaminated supplements reaching consumers (Cohen et al., 2014). Future research should include measures of a wide range of DE and weight and body change strategies and aims to model how these factors explain supplement use in both males and females. Future studies should also aim to enlighten why general adolescents consume supplements, to better understand their personal rationale for use. Finally, interventions aiming to prevent DE or to promote a positive body image in adolescents should also target supplement use and include such use as an outcome measure, especially in boys.

## Conclusion

The number of weekly supplement users was high in this sample of late adolescents, and protein and creatine supplement use was more common among boys compared to girls. Immigrant boys used more protein, creatine, and dieting supplements than their non-immigrant counterparts, and creatine supplementation was more common in immigrant girls than non-immigrant girls Use of protein, creatine, and dieting supplements was associated with some risk factors for ED development and participation in exercise and sport participation emphasizing power, leanness, and appearance in boys but not in girls. Interventions, prevention, and policy change are warranted to avoid negative health consequences from supplement use. Preventive efforts should be targeted toward boys engaged in fitness center exercise, weight class, gravitational and aesthetic sports.

## Data Availability Statement

The raw data supporting the conclusions of this article will be made available by the authors, without undue reservation.

## Ethics Statement

The studies involving human participants were reviewed and approved by Regional Committees for Medical and Health Research Ethics. Written informed consent from the participants' legal guardian/next of kin was not required to participate in this study in accordance with the national legislation and the institutional requirements.

## Author Contributions

KS-T, OF, MT, CS-B, SB-S, JR, GP, and JS-B contributed to the design and implementation of the research. KS-T, OF, and JS-B contributed to the analysis of the results. All authors contributed to the writing of the manuscript.

## Funding

This work was supported by the DAM foundation (2016/FO76521), through the Norwegian Woman's Public Health Association (H1/2016). A commercial sponsor (TINE AS) supported the study after the study protocol was published but was not involved in data collection, data analysis, or the writing of the present paper.

## Conflict of Interest

The authors declare that the research was conducted in the absence of any commercial or financial relationships that could be construed as a potential conflict of interest.

## Publisher's Note

All claims expressed in this article are solely those of the authors and do not necessarily represent those of their affiliated organizations, or those of the publisher, the editors and the reviewers. Any product that may be evaluated in this article, or claim that may be made by its manufacturer, is not guaranteed or endorsed by the publisher.
