# Supplementary material for: Protein, Creatine, and Dieting Supplements Among Adolescents: Use and Associations With Eating Disorder Risk Factors, Exercise-, and Sports Participation, and Immigrant Status
Source: Front Sports Act Living. 2021 Oct 13;3:727372. doi: 10.3389/fspor.2021.727372 (PMC8548763; doi:10.3389/fspor.2021.727372)
Supplement: Supplementary file 1 [file Table_1.DOCX]

**Supplementary file**

| Table S1: Factor structure and loadings for exercise and sport activities | | | |
| --- | --- | --- | --- |
|  | Weight sensitive sports | Aesthetical sports | General exercise and sport |
| Eigenvalue | 2.43 | 1.59 | 1.04 |
| Explained variance (%) | 24.33 | 15.88 | 10.36 |
| Strength/power sports | .710 |  |  |
| Fitness | .689 |  |  |
| Martial arts | .603 |  |  |
| CrossFit | .526 |  |  |
| Dance |  | .776 |  |
| Yoga |  | .697 |  |
| Aesthetical sports |  | .617 |  |
| Endurance exercise |  |  | .790 |
| Resistance exercise |  |  | .738 |
| Ball sports |  |  | .575 |
|  |  |  |  |
